# IMPROVE-BMT: A Pilot Randomized Controlled Trial of Prehabilitation Exercise for Adult Hematopoietic Stem Cell Transplant Recipients

**DOI:** 10.3390/jcm13072052

**Published:** 2024-04-02

**Authors:** Melanie Potiaumpai, Maxime Caru, Shin Mineishi, Seema Naik, Babette S. Zemel, Kathryn H. Schmitz

**Affiliations:** 1 Department of Medicine, School of Medicine, University of Pittsburgh, 5051 Centre Ave, Pittsburgh, PA 15213, USA; 2 Department of Pediatrics, College of Medicine, Penn State University, 500 University Drive, P.O. Box 850, Hershey, PA 17033, USA; mcaru@pennstatehealth.psu.edu; 3 Penn State Cancer Institute, College of Medicine, Penn State University, 500 University Drive, P.O. Box 850, Hershey, PA 17033, USA; smineishi@pennstatehealth.psu.edu (S.M.); snaik@pennstatehealth.psu.edu (S.N.); 4 Division of Gastroenterology, Hepatology and Nutrition, The Children’s Hospital of Philadelphia, 3401 Civic Center Blvd, Philadelphia, PA 19104, USA; 5 Department of Pediatrics, Perelmen School of Medicine, University of Pennsylvania, 3400 Civic Center Boulevard, Philadelphia, PA 19104, USA

**Keywords:** allogeneic stem cell transplant, autologous stem cell transplant, cancer, exercise, exercise oncology, oncology, pre-transplant exercise, physical function, prehabilitation

## Abstract

**Background**: There is limited evidence on the effects of aerobic and resistance training exercise interventions to improve physical function and patient-reported outcomes prior to autologous and allogeneic hematopoietic stem cell transplant (HSCT). IMPROVE-BMT was a single-site, pilot randomized controlled trial investigating the feasibility, acceptability, and safety of a pragmatic resistance training exercise program prior to HSCT compared to usual HSCT care. Secondary aims included differences in physical function between the exercise group (EX) and usual care control group (UC). **Methods**: Outcome measurements were assessed: prior to HSCT, on/around day of HSCT admission, +30 days post-HSCT, and +100 days post-HSCT. The exercise intervention was a home-based exercise program that incorporated resistance-band and bodyweight exercises. **Results**: Acceptability among participants was 83%; exercise adherence averaged at 92%; and there were zero exercise-related adverse or serious adverse events. The average pre-transplant exercise phase was 6.28 weeks (2.71–18.29 weeks). EX (*n* = 36) demonstrated larger increases in the six-minute walk test distance, short physical performance battery scores, and 30-s chair stands compared to UC (*n* = 38) and demonstrated significant within-group improvements for the six-minute walk test, the short physical performance battery, the 30-s chair stands, and the timed up-and-go test. **Conclusions**: IMPROVE-BMT demonstrates that pragmatic exercise is highly feasible for HSCT recipients and can potentially lead to enhanced recovery that may not be achievable in non-exercisers.

## 1. Introduction

The number of autologous (AUTO) and allogeneic (ALLO) hematopoietic stem cell transplant (HSCT) survivors is projected to grow to 294,000 and 234,000, respectively, by 2030 [1]. Both AUTO and ALLO HSCT recipients are at risk of developing long-term HSCT-related toxicities including reduced physical function, muscle strength, and quality of life (QoL), increased anxiety, poorer sleep quality, and long-term negative changes to body composition and bone health [2,3,4,5]. Exercise is an effective strategy peri- and post-HSCT to improve physical and psychosocial issues including cardiorespiratory function, muscle strength, physical function, fatigue, and anxiety [6,7,8,9]. Additionally, exercise has the potential to improve total and non-relapse mortality [10], hematological reconstitution, immunological capacity, and stem cell survival [9,10,11,12,13]. However, exercise during these time periods does not address the possibility of preventing HSCT-related muscular, functional, and psychosocial declines.

Prehabilitation, or prehab, takes place between cancer diagnosis and the beginning of treatment and aims to intervene and improve on physical and psychological health to reduce the incidence and severity of future treatment- and disease-related impairments [14]. Despite the growing supportive evidence of prehab exercise for other cancer sites, including breast, lung, and gastrointestinal cancer [14], there is a paucity of evidence focusing on the feasibility and efficacy of prehabilitative exercise prior to AUTO and ALLO HSCT. To address this knowledge gap and inform clinical practice, we designed the Impact of Prehabilitation in Oncology Via Exercise—Bone Marrow Transplant (IMPROVE-BMT) trial to evaluate the feasibility, acceptability, and safety of a prehabilitation exercise program in adults scheduled to receive an HSCT. We hypothesized that prehab exercise would be safe and acceptable for patients to participate in and would be feasible to complete without the risk of increased adverse events compared to usual pre-HSCT care. The secondary aims were to determine the impact of prehab exercise on objective physical function outcomes.

## 2. Materials & Methods

### 2.1. Study Design

The original study protocol has been previously published [15]. IMPROVE-BMT was a two-arm randomized controlled trial comparing an exercise intervention versus usual pre-HSCT care initiated prior to AUTO and ALLO HSCT. The Penn State Human Subjects Protection Office and Institutional Review Board approved this protocol. IMPROVE-BMT was prospectively registered at ClinicalTrials.gov (NCT03886909).

#### 2.1.1. Participants and Recruitment

Participants were adults seen at the Penn State Cancer Institute between June 2019 and August 2022 for AUTO or ALLO HSCT. Preliminary eligibility was assessed using the electronic medical record. Inclusion and exclusion criteria for participant selection have been previously published [15]. Briefly, participants had to present with a hematological malignancy in partial or complete remission, no current presentation of active infections or cytopenias, and no current evidence of an absolute contraindication to exercise (e.g., uncontrolled hypertension, cardiac insufficiency, severe musculoskeletal impairments). After receiving oncologist approval, participants were contacted and consented by the exercise and cancer specialist—a PhD-level postdoctoral fellow with multiple years of experience working with people living with and beyond cancer and held an active exercise trainer certification from the ACSM. All patients provided written consent prior to any study-related activities. Patients were stratified based on transplant type, then randomized into the Home-based exercise group (EX) or the Usual Care + Educational Program group (UC) using a random number generator in Microsoft Excel. Patients were not blinded to their group assignment. Study team members involved in patient-facing activities were also not blinded to the group assignment.

#### 2.1.2. Exercise Intervention

The pre-transplant exercise intervention is described in detail in the published protocol [15]. The pre-HSCT intervention lasted between a minimum of two weeks and a maximum of 24 weeks and was dependent on when the study participant was identified, approved for consent, and was able to complete the baseline assessments to be randomized.

The exercise intervention followed the frequency, intensity, time, and type (FITT) principle on prescribing exercise. Because this trial intended to prescribe exercise based on pragmatic principles, each exercise program was tailored for each participant’s incoming physical capabilities, clinicopathological history, and lifestyle (i.e., work schedule, caregiver responsibilities, available space, etc.). Modifications were applied to exercises if required by the patient. Each exercise session consisted of one to two warmup exercises, four to six upper- and lower-body strength exercises, followed by two cool down stretches that required 30–45 min to complete. Exercise logs were collected by the exercise and cancer specialist at clinic visits with patients and used to calculate exercise adherence and as a guide on if/how to modify future exercise sessions.

#### 2.1.3. Usual Care

UC patients received an educational counseling session that included information on HSCT precautions and expectations, and post-HSCT exercise recommendations. At the end of the study, participants were offered a no-cost exercise counseling session with an exercise and cancer specialist.

#### 2.1.4. Outcomes

Primary outcomes: Acceptability was defined as the proportion of approached patients who agreed to participate and complete at least the prehab counseling session. The intervention was considered feasible if 50% of the included patients completed at least 1/3 of the prescribed exercise sessions for 2 weeks or more. Safety was reported by the number of exercise-related adverse or serious adverse events. Exercise adherence was calculated as the proportion of completed exercise sessions over the number of prescribed exercise sessions.

Secondary outcomes: Outcome measurements were assessed at: baseline (t_0_), on/around day of HSCT admission (t_1_), +30 days post-HSCT (t_2_), and +100 days post-HSCT (t_3_). Physical function assessments included the six-minute walk test (6MWT) [16,17], the 30-s chair stand (30CST) [18], the timed up-and-go (TUG) [19], the short physical performance battery (SPPB) [20,21], the Berg balance scale [22,23,24], and isometric handgrip strength [25,26], further described in the published study protocol [15]. We also retrospectively collected the following clinical outcomes pertaining to healthcare services use: hospital duration, engraftment period, readmission within +30 days and +100 days post-HSCT, unexpected emergency department admissions, and one-year post-HSCT survival.

## 3. Statistical Analysis

Patient characteristics and clinical outcomes were summarized by randomization groups. The primary outcomes of acceptability, adherence, and safety were reported as frequencies and proportions. Statistical significance was defined a priori as *p* < 0.05, two-sided. Data collected for the functional measures were analyzed using linear mixed-effect models for repeated measures, using maximum likelihood estimation to produce group mean estimates and within-group and between-group differences for each measurement point, with 95% confidence intervals. All analyses were performed using SPSS 29.0 (IBM Corp., Armonk, NY, USA).

## 4. Results

Of the 82 adults who consented, eight were screen failures, so the final analysis included 74 participants (Table 1). Participants were on average 60 years old, 35% women, the majority were Caucasian (74%), and the majority presented with three or more comorbidities at the study start. Of 82 participants, 27 were diagnosed with multiple myeloma (32.9%), 18 were diagnosed with AML (22.0%), 9 were diagnosed with MDS (11.0%), and the remaining participants were diagnosed with a variety of lymphomas and leukemias.

### Primary Outcomes

**Figure 1:**
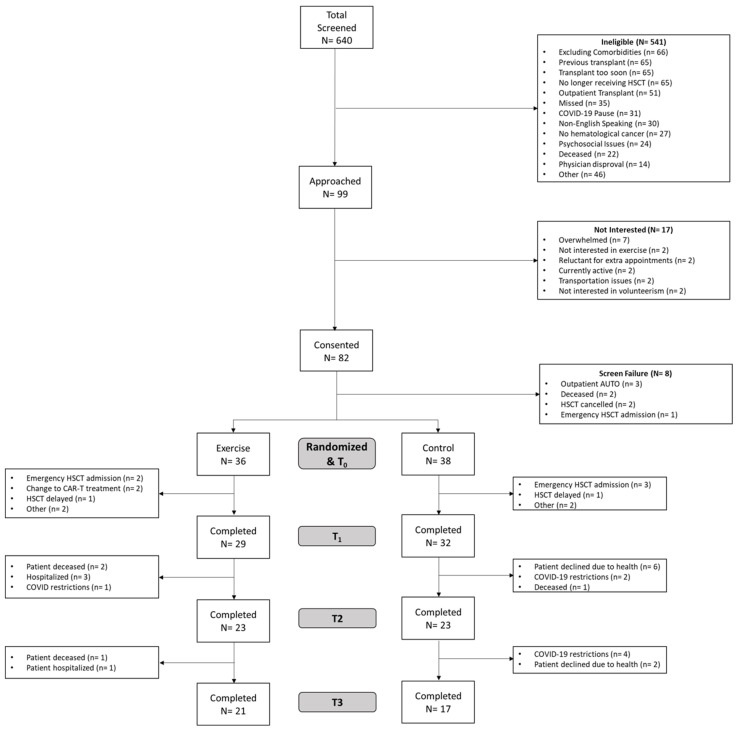
CONSORT of participants in study flow.

Recruitment took place between June 2019 and September 2022 with a total of nine non-consecutive months of COVID-19-related pauses in recruitment and study-related activities. Figure 1 presents a CONSORT flow diagram. Between June 2019 and September 2022, 640 adults were screened for potential eligibility—541 were deemed ineligible (84%). The leading reasons for ineligibility included: (1) excluding comorbidity (*n* = 66, 12%); (2) previous HSCT (*n* = 65, 12%); (3) scheduled transplant < 2 weeks (*n* = 65, 12%); (4) no longer scheduled to undergo HSCT (*n* = 65, 12%); and (5) outpatient AUTO transplant (*n* = 51, 9%). The remaining reasons are listed in Figure 1. Of the remaining 99 potential participants approached for the study, 17 declined participation (17%), and 82 consented, resulting in an 83% acceptability rate. Of the 82 patients who consented to participate, eight were screen failures, and 74 were randomized to EX (*n* = 36) or UC (*n* = 38). In the EX group, seven patients withdrew prior to the end of prehab (t_1_) due to early emergency HSCT admission, a change in treatment, or other undisclosed reasons. Prior to +30 days HSCT, six more participants withdrew due to passing away, being hospitalized for HSCT-related complication, or because of COVID-related patient restrictions. Prior to +100 days HSCT, one participant passed away due to malignancy-related complications and one participant was hospitalized for HSCT-related complications. Three participants passed away during the duration of the study due to non-study-related reasons: (1) an HSCT-related complication (*n* = 1); (2) acute graft-versus-host disease (*n*= 2); and (3) a malignancy-related complication (*n* = 1).

Of the 36 patients randomized to EX, 86% (*n* = 31) completed 1/3 of the prescribed exercise session prescribed for 2 weeks or more, deeming the intervention as feasible based on our a priori definition. There were 0 (0%) exercise-related adverse or serious adverse events reported on the patient-reported outcome surveys or to the study interventionist.

## 5. Intervention Duration

For all patients in EX, the average duration of the pre-transplant exercise phase was 6.28 weeks (range: 2.71–18.29 weeks). For ALLO recipients, prehab lasted 6.99 weeks (range: 2.71–18.29 weeks). For AUTO recipients, prehab lasted 5.52 weeks (range: 2.71–12.71 weeks).

## 6. Exercise Adherence

Of the 36 patients in EX, 31 returned exercise logs (86% return rate). Overall, exercise adherence across all patients, regardless of transplant type, was high at an average of 91.98% (SD = 34.59), ranging from 33% to 190%. When separated by transplant type (AUTO versus ALLO), exercise adherence did not greatly differ. For ALLO recipients, the average exercise adherence was 92.99% (SD = 42.48), ranging from 33% completion to 190%. For AUTO recipients, the average exercise adherence was 90.89% (SD = 25.04), ranging from 55% to 131%.

## 7. Secondary Outcomes

### AUTO versus ALLO Differences

Table 2 presents differences between AUTO and ALLO HSCT recipients in EX. There were no significant differences in the performed functional assessments between AUTO and ALLO HSCT at any study time points among EX participants. At +100 days post-HSCT, the AUTO group showed a trend towards enhanced recovery compared to the ALLO group for the 6MWT (mean difference (MD) = 99.20, 95% CI (−12.01, 210.40), *p* = 0.08) and the 30CST (MD = 3.42, 95% CI (−0.50, 7.33), *p* = 0.09).

Table 3 and Table 4 present mean estimates and between- and within-group differences, respectively, for changes in the physical function outcomes. For the 6MWT, marginally greater improvements were seen in the EX group versus the UC group at T_1_ after prehab (MD = 49.72, 95% CI (−6.05, 105.49), *p* = 0.08) (Table 3). The distance walked at T_3_ in the EX group was also marginally significantly higher compared to the UC group (MD = 62.82, 95% CI (−10.25, 135.88), *p* = 0.09) (Table 3). Despite a significant decrease from T_1_ to T_2_ in the EX group (MD = −101.85, 95% CI (21.48, 184.22), *p* = 0.007), the EX group was able to significantly improve the distance (Table 2) walked at T_3_ (MD = 131.21, 95% CI (41.02, 221.40), *p* = 0.001) and surpassed the initial distance at baseline (MD = 24.98 m, 95% CI (−32.20, 82.15), *p* = 1.00) (Table 4). The UC group exhibited a similar pattern of change in distance walked; however, the magnitude of changes was not significant and the UC group was unable to improve the overall distance walked over the length of the study and was unable to match their baseline aerobic capacity value (MD = −11.67, 95% CI (−73.15, 49.82), *p* = 1.00) (Table 4).

For the SPPB, the EX group saw greater improvements in the total score after prehab exercise (MD = 0.88, 95% CI (−0.18, 1.93), *p* = 0.10) compared to the UC group, and sustained improvements at +100 days post-HSCT compared to the UC group (MD = 1.11, 95% CI (0.11, 2.09), *p* = 0.03) (Table 3). Only the EX group showed overall improvement (T_3_) with a 0.90 point (95% CI (0.08, 1.71, *p* = 0.02)) increase compared to baseline (T_0_) (Table 4).

For the 30-s chair stands, the UC group demonstrated an overall decrease in lower body strength (MD = −1.87, 95% CI (−4.37, 0.63), *p* = 0.27) over the length of the study and performed significantly worse at +100 days post-HSCT compared to the EX group (MD = 3.79, 95% CI (0.74, 6.84), *p* = 0.02) (Table 3). The EX group demonstrated improved lower body strength after prehab exercise (MD = 1.69, 95% CI (0.41, 2.98), *p* = 0.004) and despite a significant decrease +30 days post-HSCT (MD = −5.22, 95% CI (−8.20, −2.24), *p* < 0.001), the EX group significantly increased the number of chair stands completed at +100 days post-HSCT (MD = 5.37, 95% CI (1.93, 8.81), *p* < 0.001) with an overall increase above baseline at +100 days post-HSCT (MD = 1.84, 95% CI (−0.42, 4.10), *p* = 0.18) (Table 4).

There were no significant differences or trends for isometric handgrip strength or for the Berg Balance Test for either the EX or UC groups at any timepoints over the length of the study.

Table 5 illustrates select clinical outcomes and health services use between the EX and UC groups. Hospital duration was similar between the EX and UC groups, ranging from ~1.4 weeks to 9 weeks. The engraftment period, following HSCT infusion, was similar between the EX and UC groups at an average of ~16 days, ranging from 9–33 days to achieve HSCT engraftment. There were also no differences in the proportion of patients in the EX and UC groups that were unexpectedly readmitted within +30 days post-HSCT and +100 days post-HSCT. Further, the number of emergency department admissions was not different between the EX and UC groups. Finally, one-year survival did not differ between the EX and UC groups.

## 8. Discussion

IMPROVE-BMT found that patients are eager to participate in an exercise program prior to AUTO or ALLO HSCT, show high adherence to the prescribed exercise program, and exercise in this high-risk population is safe. This trial also demonstrates strong preliminary efficacy of pre-HSCT exercise on improving functional capacity and enhanced functional recovery post-HSCT. EX patients showed improvements in aerobic capacity, physical function, and lower extremity strength and power over the length of the study despite experiencing significant declines at +30 days post-HSCT and showed overall improvements that surpassed their initial baseline functionality. UC showed no functional changes prior to HSCT and over the length of the study showed declines in aerobic capacity, lower extremity strength, and power and balance that did not match or surpass their initial baseline functionality.

Our acceptability rate of 83% was high compared to previous trials, which ranged from 20% to 63% [27,28,29,30]. The exclusion of a large proportion of potential HSCT recipients (*n* = 541) is not uncommon given the leading reasons behind ineligibility—excluding comorbidity, previous HSCT, insufficient intervention time, or cancelled HSCT. These exclusions provide an opportunity for future trials to expand the number of potential participants by tailoring the prehab exercise to target comorbidities susceptible to exercise-induced changes, which may increase the potential to receive future treatment options, including HSCT. Further, future prehab trials will be able to find ways to identify participants at an earlier stage of the HSCT workup process to allow for more time for prehab.

Exercise adherence in our trial was high (91.98%, range: 33–190%), regardless of transplant. Previous reports of exercise adherence have ranged from 55% to 99% [29,30,31,32,33], while other trials were unable to report on exercise adherence due to failure of study participants to return exercise logs [28]. The high exercise adherence rate seen in this trial could be attributed to the pragmatic design of the exercise program. Each participants’ exercise program was modified based on their baseline functional performance, existing comorbidities and physical activity levels, and lifestyle factors (e.g., employment status, caregiver responsibilities, exercise equipment ownership). Allowance for these accommodations allows exercise to fit the patient’s lifestyle, rather than making a patient’s lifestyle fit the exercise program, mimicking “real world” exercise habits. Further, there were zero intervention-related adverse or serious adverse events, consistent with previous trials investigating pre-HSCT exercise, regardless of transplant and hematologic malignancy type [28,29,30,32,33]. This further supports the assertion that if exercise is pragmatically programed and prescribed to accommodate the patients clinicopathologic history while also targeting functional improvements, exercise is safe to be performed unsupervised for a large proportion of AUTO and ALLO HSCT recipients.

The secondary functional outcomes explored in this trial demonstrated strong promise for exercise prior to HSCT. The most noteworthy changes were seen in the six-minute walk distance. Compared to UC, the EX increased their 6MWT, surpassing the range of 14.0 to 30.5 m considered to be minimal for an important different [34,35]. In addition, at +100 days post-HSCT, EX demonstrated enhanced recovery in aerobic capacity by surpassing their initial baseline values, aligning with previous reports [28,30,32,33]. Improving cardiovascular function and aerobic capacity in HSCT has been established as a high priority, as cardiovascular disease and cardiovascular complications are one of the most common complications associated with HSCT [36]. The complications occur acutely (within +100 days post-HSCT) and chronically, years after transplantation, increasing the risk for cardiovascular disease in HSCT survivors by a minimum of fourfold compared to the general population [36,37]. The average duration of participation in our trial was approximately six weeks, demonstrating the potent effect exercise has on improving aerobic capacity prior to HSCT, and its potential effect for prolonged enhanced recovery with longer prehab participation. Conversely, changes in clinical and health services use showed no difference between the EX and UC groups. This is not unexpected as the duration of the prehab intervention was not long enough and did not extend past HSCT admission to elicit any significant clinical changes past the prehab period. Given the positive findings in feasibility, acceptability, and safety of the intervention, a future larger trial could incorporate these variables and additional HSCT-related outcomes.

To our knowledge, there are only a few published trials that also included both AUTO and ALLO HSCT [29,32]. Previous trials have targeted one type of transplant and/or focused on one type of hematologic malignancy. The decision to include patients regardless of transplant and disease type was intentional to create a more pragmatic study design that reflects the practicalities of working in a clinical oncology setting and what an exercise and cancer specialist might encounter when working with an HSCT clinic. Functional changes seen in EX were not impacted by the inclusion of both AUTO and ALLO. Over the length of the study, AUTO and ALLO EX group participants showed similar patterns of functional decline and improvements. The trending differences seen between AUTO versus ALLO at +100 days post-HSCT for the 6MWT and 30CST may likely be due to differences in the type and severity of symptomology. Previous evidence has shown that ALLO recipients display suppressed cardiovascular function and lengthened recovery time compared to AUTO HSCT [38], indicating that ALLO HSCT recipients may require a more aerobic-centric exercise prescription to enhance pre-ALLO cardiovascular function and enhanced recovery that could potentially match AUTO HSCT. The 30CST serves as a surrogate for lower-body strength and power [18,39]. ALLO recipients are commonly placed on long-term corticosteroid use for the prevention and control of acute and chronic graft-versus-host disease. A well-known side effect of long-term corticosteroid use is corticosteroid-induced myopathy, causing progressive weakness and a reduced ability to use the proximal muscles of the upper and lower limbs. The difference in magnitude of recovery between AUTO versus ALLO could have resulted from undiagnosed and untreated corticosteroid-induced myopathy. This has also been reported by Morishita et al., who demonstrated that the total corticosteroid dose was significantly correlated with decreased knee extensor strength but not significantly correlated with 6MWT performance [40]; and by Ngo-Huang et al., who reported a significant negative association between cumulative corticosteroid dose and 6MWT distance, hip flexor strength, and knee extensor strength [41]. Future efforts should be made to identify corticosteroid-induced myopathy early and design the exercise program to help mitigate any effects.

A strength of the IMPROVE-BMT trial was that it was designed to be pragmatic to accommodate patients’ needs and abilities. However, this approach has its limitations including that it may be more difficult to implement in institutions and organizations who do not have a specially trained exercise and cancer specialist and need to rely on a standardized program for ease of use. Another limitation of IMPROVE-BMT was the number of participants who were considered ineligible for various reasons. To be truly pragmatic, future efforts should be made to create processes that work within the clinic workflow and widen the involvement of HSCT healthcare team members to identify potential patients as early as possible to ensure the possibility of pre-HSCT exercise and other necessary pre-transplant support services.

## 9. Conclusions

In summary, we found that pre-transplant exercise is highly acceptable and feasible for patients to participate in and complete. Pre-HSCT exercise is safe and demonstrates early effectiveness at promoting elevated increases in functionality post-AUTO and ALLO HSCT. The reported functional data suggest that adults with hematologic malignancies scheduled for HSCT may highly benefit from engaging in tailored exercise prior to HSCT to mitigate forthcoming functional declines and aid in long-term functional recovery and preservation.

## Figures and Tables

**Table 1:** Participant characteristics.

	Overall (*n* = 82)	Exercise (*n* = 36)	Usual Care (*n* = 38)
Age (years)	60.71 ± 12.12	57.30 ± 15.55	63.47 ± 7.70
Sex			
Female	29 (35.37)	16 (44.4)	12 (31.6)
Male	53 (64.63)	20 (55.6)	26 (68.4)
Race			
White or Caucasian	74 (90.2)	32 (88.9)	35 (92.1)
Black or African American	6 (7.3)	3 (8.3)	2 (5.3)
Asian	2 (2.4)	1 (2.7)	1 (2.6)
Ethnicity			
Not Hispanic or Latino	81 (98.8)	35 (97.2)	38 (100)
Hispanic or Latino	1 (1.2)	1 (2.7)	0 (0)
Transplant			
AUTO	42 (51.22)	18 (50.0)	19 (50)
ALLO	40 (48.78)	18 (50.0)	19 (50)
Height (cm)	169.61 ± 8.47	169.01 ± 8.40	169.23 ± 8.71
Weight (kg)	84.78 ± 20.61	85.49 ± 19.83	81.85 ± 21.02
Body Mass Index (BMI)	29.28 ± 5.93	29.81 ± 6.00	28.35 ± 5.95
Hematopoietic Cell Transplantation-specific Comorbidity Index (HCT-CI)			
0	7 (8.5)	3 (8.3)	4 (10.5)
1–2	15 (18.3)	10 (27.8)	5 (13.2)
3+	38 (46.3)	14 (38.9)	24 (63.2)
Missing	22 (26.8)	9 (25.0)	5 (13.2)
Cancer Type			
Multiple Myeloma	27 (32.9)	9 (25.0)	14 (36.8)
Acute Myeloid Leukemia	18 (22.0)	6 (16.6)	11 (28.9)
Myelodysplastic Syndrome	9 (11.0)	6 (16.6)	2 (5.3)
Diffuse Large B Cell Lymphoma	6 (7.3)	4 (11.1)	1 (2.6)
Mantle Cell Lymphoma	4 (4.9)	0 (0)	4 (10.5)
Chronic Myelogenous Leukemia	3 (3.7)	2 (5.6)	3 (7.9)
Other Leukemias	7 (8.5)	5 (13.9)	1 (2.6)
Other Lymphomas	6 (7.3)	2 (5.6)	2 (5.3)
Other Malignancies	2 (2.4)	2 (5.6)	0 (0)
Hospital Duration (weeks)	3.45 ± 1.32 Range: 1.43–9.29	3.56 ± 1.46 Range: 1.43–9.29	3.37 ± 1.21 Range: 2.00–7.00

Values are mean ± SD; # (%).

**Table 2:** Physical function outcomes: exercise group: AUTO vs. ALLO.

	AUTO		ALLO		*p*-Value Mean Difference (95% CI)
Outcome	Estimate	95% CI	Estimate	95% CI	
Six-Minute Walk Test (m)					
T0	421.70	373.59, 469.80	428.69	380.59, 476.80	0.84 −6.99 (−75.03, 61.03)
T1	429.67	375.72, 483.62	413.51	359.99, 467.03	0.67 16.16 (−59.84, 92.15)
T2	306.45	225.28, 387.61	332.05	232.82, 431.27	0.69 −25.60 (−153.75, 102.55)
T3	498.56	419.93, 577.20	399.36	320.73, 477.99	0.08 99.20 (−12.01, 210.40)
SPPB (Total Score)					
T0	10.56	9.77, 11.34	10.28	9.49, 11.06	0.62 0.28 (−0.83, 1.39)
T1	11.06	10.31, 11.80	11.17	10.44, 11.90	0.82 −0.12 (−1.16, 0.93)
T2	10.05	8.95, 11.16	10.24	8.88, 11.60	0.83 −0.19 (−1.94, 1.56)
T3	11.64	10.83, 12.45	10.99	10.17, 11.81	0.26 0.65 (−0.50, 1.80)
30-Second Chair Stands (#)					
T0	14.03	11.82, 16.23	13.81	11.60, 16.01	0.89 0.22 (−2.90, 3.34)
T1	16.18	13.85, 18.52	15.08	12.77, 17.39	0.50 1.10 (−2.18, 4.39)
T2	10.86	6.86, 14.86	9.88	5.18, 14.59	0.75 0.98 (−5.20, 7.15)
T3	17.43	14.70, 20.17	14.02	11.22, 16.81	0.09 3.42 (−0.50, 7.33)
Timed Up-and-Go (sec)					
T0	10.74	9.17, 12.32	11.18	9.60, 12.76	0.70 −0.43 (−2.67, 1.80)
T1	9.44	7.68, 11.21	10.27	8.52, 12.01	0.51 −0.82 (−3.31, 1.66)
T2	12.11	9.29, 14.93	13.99	10.68, 17.31	0.38 −1.89 (−6.24, 2.47)
T3	9.79	7.61, 11.97	10.72	8.48, 12.96	0.55 −0.93 (−4.05, 2.20)
Handgrip Strength (kg)					
T0	24.35	20.09, 28.61	28.42	24.16, 32.68	0.18 −4.07 (−1.09, 1.95)
T1	25.76	21.35, 30.16	29.33	24.99, 33.68	0.25 −3.57 (−9.76, 2.62)
T2	23.95	19.62, 28.27	24.76	20.24, 29.27	0.80 −0.81 (−7.06, 5.44)
T3	23.50	18.87, 28.13	24.67	19.99, 29.35	0.72 −1.17 (−7.75, 5.42)
Berg Balance Score (Total Score)					
T0	52.00	49.91, 54.10	54.28	52.18, 56.37	0.13 −2.28 (−5.24, 0.68)
T1	52.99	50.13, 55.84	54.66	51.87, 57.45	0.38 −1.67 (−5.67, 2.32)
T2	51.23	48.47, 53.99	53.05	49.87, 56.23	0.38 −1.82 (−6.03, 2.40)
T3	53.51	50.82, 56.19	53.45	50.60, 56.30	0.98 0.06 (−3.86, 3.97)

Mean difference: AUTO–ALLO.

**Table 3:** Physical function outcomes: between-group differences.

	Exercise		Usual Care		*p*-Value Mean Difference (95% CI)
Outcome	Estimate	95% CI	Estimate	95% CI	
Six-Minute Walk Test, m					
T0	425.20	388.23, 462.16	399.02	363.04, 434.99	0.32 26.18 (−25.40, 77.76)
T1	421.81	381.77, 461.85	372.09	333.27, 410.92	0.08 49.72 (−6.05, 105.49)
T2	318.96	253.96, 383.97	316.56	251.94, 381.17	0.96 2.41 (−89.25, 94.06)
T3	450.17	399.59, 500.75	387.35	334.63, 440.08	0.09 62.82 (−10.25, 135.88)
SPPB, Total Score					
T0	10.42	9.56, 10.75	10.16	9.56, 10.75	0.55 0.26 (−0.59, 1.11)
T1	11.11	10.35, 11.87	10.23	9.50, 10.97	0.10 0.88 (−0.18, 1.93)
T2	10.04	8.92, 11.17	9.09	7.97, 10.22	0.24 0.95 (−0.64, 2.54)
T3	11.31	10.63, 11.99	10.22	9.50, 10.94	0.03 1.11 (0.11, 2.09)
30-s Chair Stands					
T0	13.92	12.42, 15.42	13.84	12.38, 15.30	0.94 −0.08 (−2.02, 2.17)
T1	15.61	13.98, 17.24	13.86	12.28, 15.43	0.13 1.76 (−0.51, 4.02)
T2	10.39	7.91, 12.87	10.79	8.29, 13.30	0.82 −0.40 (−3.93, 3.12)
T3	15.76	13.66, 17.86	11.97	9.76, 14.19	0.02 3.79 (0.74, 6.84)
Timed Up-and-Go (sec)					
T0	10.96	9.88, 12.05	10.95	9.89, 12.01	0.99 0.01 (−1.51, 1.53)
T1	9.85	8.65, 11.04	10.22	9.06, 11.39	0.66 −0.37 (−2.04, 1.29)
T2	12.91	11.17, 14.64	11.17	9.38, 12.95	0.17 1.74 (−0.75, 4.23)
T3	10.24	8.83, 11.66	10.69	9.21, 12.17	0.67 −0.45 (−2.50, 1.60)
Handgrip Strength (kg)					
T0	26.39	23.23, 29.54	26.08	23.01, 29.15	0.89 0.31 (−4.09, 4.71)
T1	27.54	24.36, 30.72	25.58	22.49, 28.67	0.38 1.96 (−2.47, 6.40)
T2	24.63	21.16, 28.11	22.58	19.14, 26.02	0.41 2.05 (−2.84, 6.94)
T3	24.13	20.71, 27.56	25.17	21.70, 28.64	0.67 −1.04 (−5.91, 3.84)
Berg Balance Score					
T0	53.14	51.68, 54.60	52.79	51.37, 54.21	0.73 0.35 (−1.69, 2.38)
T1	53.82	52.20, 55.44	52.40	50.83, 53.97	0.21 1.43 (−0.83, 3.68)
T2	52.12	47.93, 56.32	48.38	44.09, 52.67	0.22 3.75 (−2.25, 9.75)
T3	53.75	51.84, 55.66	52.29	50.24, 54.34	0.30 1.46 (−1.34, 4.26)

Mean difference: intervention–control.

**Table 4:** Physical function outcomes: within-group differences.

	Exercise			Usual Care		
Outcome	Estimate	95% CI	*p*-Value	Estimate	95% CI	*p*-Value
Six-Minute Walk Test, m						
T0 to T1	+3.39	−33.63, 40.39	0.26	+6.93	−8.79, 62.65	0.26
T1 to T2	−102.85	21.48, 184.22	0.007	−55.54	−136.92, 25.84	0.39
T2 to T3	+131.21	41.02, 221.40	0.001	+70.80	−21.63, 163.22	0.24
T0 to T3	+24.98	−32.20, 82.15	1.00	−11.67	−73.15, 49.82	1.00
SPPB, Total Score						
T0 to T1	+0.69	−0.24, 1.62	0.28	+0.08	−0.83, 0.97	1.00
T1 to T2	−1.11	−2.34, 0.50	0.40	−1.14	−2.70, 0.42	0.30
T2 to T3	+1.27	−0.22, 2.76	0.14	+1.12	−0.39, 2.63	0.28
T0 to T3	+0.90	0.08, 1.71	0.02	+0.06	−0.82, 0.94	1.00
30-s chair Stands						
T0 to T1	+1.69	0.41, 2.98	0.004	+0.01	−1.23, 1.25	1.00
T1 to T2	−5.22	−8.20, −2.24	<0.001	−3.06	−6.11, −0.01	0.05
T2 to T3	+5.37	1.93, 8.81	<0.001	+1.18	−2.45, 4.81	1.00
T0 to T3	+1.84	−0.42, 4.10	0.18	−1.87	−4.37, 0.63	0.27
Timed Up-and-Go						
T0 to T1	−1.11	0.10, 2.13	0.02	−0.73	−1.72, 0.26	0.30
T1 to T2	+3.06	−5.16, −0.97	0.001	+0.95	−1.26, 3.15	1.00
T2 to T3	−2.67	−4.98, −0.35	0.02	−0.48	−2.96, 2.01	1.00
T0 to T3	−0.72	−2.20, 0.76	1.00	−0.26	−1.90, 1.37	1.00
Handgrip Strength						
T0 to T1	+1.16	−1.02, 3.34	0.91	−0.50	−2.60, 1.60	1.00
T1 to T2	−2.91	−5.71, −0.12	0.04	−3.00	−5.88, −0.13	0.04
T2 to T3	−0.50	−3.75, 2.76	1.00	2.59	−0.87, 6.05	0.27
T0 to T3	−2.25	−5.06, 0.55	0.19	−0.91	−3.95, 2.12	1.00
Berg Balance Score						
T0 to T1	+0.68	−2.96, 1.74	1.00	−0.39	−2.19, 1.41	1.00
T1 to T2	−1.70	−7.37, 3.97	1.00	−4.02	−9.83, 1.79	0.37
T2 to T3	1.63	−4.16, 7.41	1.00	3.91	−2.08, 9.90	0.47
T0 to T3	0.61	−1.74, 2.96	1.00	−0.50	−3.08, 2.08	1.00

T0: baseline; T1: post-prehab, pre-HSCT; T2: +30 days HSCT; T3: +100 days HSCT.

**Table 5:** Clinical outcomes.

	Exercise	Usual Care
Hospital Duration (Weeks)	3.56 ± 1.46 Range: 1.43–9.29	3.37 ± 1.21 Range: 2.00–7.00
Engraftment Period (Days)	16.63 ± 7.01 Range: 9–33	16.42 ± 6.31 Range: 9–33
30-Day Readmission		
No readmission	27 (90)	30 (88.2)
Readmission	3 (10)	4 (11.8)
100-Day Readmission		
No readmission	23 (76.7)	24 (70.6)
Readmission	7 (23.3)	10 (29.4)
ED Admissions (#)		
0	23 (76.7)	23 (67.6)
1–2	5 (16.7)	11 (32.4)
3+	2 (6.6)	0 (0)
One-Year Survival		
Survived	25 (83.3)	30 (88.2)
Deceased	5 (16.7)	4 (11.8)

Values are mean ± SD; # (%).

## Data Availability

The datasets generated during and/or analyzed during the current study are available from the corresponding author on reasonable request.
